# The Relationship between Clinical Feature, Complex Immunophenotype, Chromosome Karyotype, and Outcome of Patients with Acute Myeloid Leukemia in China

**DOI:** 10.1155/2015/382186

**Published:** 2015-04-07

**Authors:** Bingjie Ding, Lanlan Zhou, Xuejie Jiang, Xiaodong Li, Qingxiu Zhong, Zhixiang Wang, Zhengshan Yi, Zhongxin Zheng, Changxin Yin, Rui Cao, Libin Liao, Fanyi Meng

**Affiliations:** ^1^Hematology Department, Nanfang Hospital, Southern Medical University, Guangzhou 510515, China; ^2^Hematology Department, Kanghua Hospital, Dongguan 523080, China

## Abstract

Mixed phenotype acute leukemia (MPAL) is a complex entity expressing both lymphoid and myeloid immunophenotyping. In the present study, 47 MPAL, 60 lymphoid antigen-positive acute myeloid leukemia (Ly^+^AML), and 90 acute myeloid leukemia with common myeloid immunophenotype (Ly^−^AML) patients were investigated. We found that, in MPAL patients, there were high proportions of blast cells in bone marrow and incidence of hepatosplenomegaly, lymphadenopathy, and Philadelphia chromosome. The overall survival (OS) and relapse-free survival (RFS) in MPAL patients were significantly shorter than those in Ly^+^AML and Ly^−^AML. With regard to the patients with normal karyotype only, the OS and RFS of MPAL were significantly lower than those of the Ly^+^AML and Ly^−^AML; but there were no significant differences in OS and RFS among the patients with complex karyotype. The OS rates of 3 groups with complex karyotype were lower than those of patients with normal karyotype. In Cox multivariate analysis, complex karyotype was an independent pejorative factor for both OS and RFS. Therefore, MPAL is confirmed to be a poor-risk disease while Ly^+^AML does not impact prognosis. Complex karyotype is an unfavorable prognosis factor in AML patients with different immunophenotype. Mixed immunophenotype and complex karyotype increase the adverse risk when they coexist.

## 1. Introduction

Acute leukemia (AL) is usually classified as myeloid or lymphoid according to the morphologic, antigenic, cytogenetic, and molecular profile of the blasts. However, close to 2 to 5% of cases [1, 2], the lineage of blast cells is not clear because both of the expression of lymphoid and myeloid immunophenotyping can be detected. In 1995, criteria for the diagnosis of AL with biphenotypic marker were established by the European Group for the Immunological Characterization of Leukemia (EGIL) explicitly [3], and then this scoring system was improved for AL with ambiguous lineages in 1998. This scoring system is based on the immunological markers including myeloid or T/B lymphoid blasts specifically to classify biphenotypic AL and clinical outcome measurements for many years. In these criteria, new mixed phenotype acute leukemia (MPAL) diagnostic classification was established for the clinic treatment and research of this disease. According to this criteria, the term of MPAL (or biphenotypic or true mixed acute leukemia) refer to acute leukemias containing the two lineages with specific antigens scores higher than 2 in more than one lineage regardless whether one or more than one population of blasts is seen. Moreover, in Ly^+^AML myeloid blasts coexpress lymphoid antigens but lymphoid antigen score is less than 2 points.

Hitherto, although lack of the MPAL cases research according to the WHO 2008 classification was reported, it is well known that MPAL appears to be a complex entity with different biological characters [4] and low survival rates [5]. Furthermore, following studies demonstrated that expressing both myeloid and lymphoid markers have been regarded as a negative prognostic factor in AL [6, 7]. To date, randomized studies about outcome in Ly^+^AML patients have led to inconsistent conclusions and there is lack of reports comparing clinic characteristic and outcome in MPAL with Ly^+^AML. As the result, the therapy for this subtype of leukemia is not consistent. The goal of present study was to analyze the molecular genetic, cytogenetic, and immunophenotypic features of patients with MPAL according to WHO 2008 classification and the relationship between clinic outcomes and laboratory characteristics.

## 2. Materials and Methods

### 2.1. Patients

A total of 197 patients with acute myeloid leukemia diagnosed according to WHO 2008 criteria [8], who were hospitalized at the Nanfang Hospital (Guangzhou, China) between January 2002 and October 2013, were retrospectively enrolled in this study. Of these, 47 were patients with mixed immunophenotype acute leukemia (MPAL), 60 were with lymphoid antigen-positive acute myeloid leukemia (Ly^+^AML), and 90 were with acute myeloid leukemia with common myeloid immunophenotype (Ly^−^AML). They were matched based on the subtypes, gender, and age (Table 1). AML after myelodysplastic syndrome (MDS) or chronic myeloid leukemia (CML) and cases being PML/RAR*α*-positive were excluded. Cases were classified by morphology and simultaneously by cytochemistry for myeloperoxidase (POX), sodium fluoride sensitive naphthalene acetic acid esterase (NAE/NAF), and glycogen (PAS). Study protocol underwent thorough review and approval process at the hospital's ethics committee. Informed consent was obtained from all patients included in the study.

### 2.2. Immunophenotyping

FCM immunophenotyping was performed on isolated bone marrow mononuclear cells by flow cytometry after collection according to standard procedure. All cases were profiled by a panel of leukocyte-associated markers, including cMPO, CD117, CD13, CD33, CD14, CD15, CD64, and CD11b/c for the myeloid lineage; cyCD3, CD2, CD5, CD7, and CD8 for T-cell lineage, cyCD79a, CD22, CD19, CD10, CD22, and CD20 for B-cell linage; and CD34 and HLA-DR for the stem/progenitor cell clone. A marker was considered positive when more than 20% of the blasts showed a positive signal.

### 2.3. Cytogenetics

Conventional cytogenetic analysis was carried out on direct preparations or 24 h unstimulated culture of bone marrow cells according to standard technique of G-banding. Meanwhile, the abnormal cloning was defined based on the criteria from International System for Cytogenetic Nomenclature (ISCN). FISH studies aimed at detecting AML1/ETO, PML/RARa, CBF*β*/MYHll, BCR/ABL, and MLL rearrangements as well as P53, Del(5q), Del(7q), Del(20q), and +8 were performed according to manufacturer's instructions.

### 2.4. Treatment Protocols

Patients who received at least 2 circles of chemotherapy were involved in current study in order to analyze clinic outcome. All patients received induction and maintenance treatment according to guidelines set by the Hematological Society of the Chinese Medical Association. The induction treatments were as follows: ① combined AML/ALL regimens (DOALP and DOAP), ② ALL-type induction therapy (VDLP and VDCP), and ③ AML-type induction therapy (DA/DAE/HA/TA). Meanwhile, they received postremission therapy as follows: ④ 3 circles of consolidation therapy and less than 3 circles of MD-Ara-C/HD-MTX, ⑤ equal to or more than 3 circles of MD-Ara-C/HD-MTX or more than 3 circles of Hyper-CVAD-A/B or autotransplantation after 5 median circles of standard-dose induction therapy, which ranges from 3 to 10 circles, and ⑥ allogeneic hematopoietic stem cell transplantation after 3 to 6 circles of consolidation or intensive therapy. The distribution of cases of induction regimen and consolidation therapy was summarized in Table 2.

### 2.5. Statistical Analysis

The two independent samples nonparametric tests were used for comparison of quantitative data. Kaplan-Meier method was used to estimate the long survival of patients. Cox univariate and multivariate analyses were performed to identify risk factors related to OS and RFS. The Pearson *χ* ^2^ tests were used for the analysis of qualitative data. All analyses were done with SPSS version 17.0, and *P* value of less than 0.05 was considered to be associated with statistical significance.

## 3. Results

### 3.1. Patients

Patient characteristics were summarized in Table 1. We identified 47 new patients with MPAL which was diagnosed using the WHO 2008 classification. Compared with Ly^+^AML or Ly^−^AML patients, MPAL patients displayed a higher ratio of lymphadenopathy and hepatosplenomegaly (63.8%) and higher percentage of blasts in bone marrow (67.6%). However, there were no statistical differences between the two groups of Ly^+^AML and Ly^−^AML.

### 3.2. Immunophenotype

Immunophenotype data showed that the positive incidence of CD34 in MPAL (76.6%) and Ly^+^AML (81.7%) subtypes was significantly higher than in Ly^−^AML group (47.8%). On the contrary, a marker of differentiated cells, CD117, was significantly lower in MPAL cases (8.5%) than Ly^+^AML (61.7%) and Ly^−^AML (51.1%).

### 3.3. Cytogenetics

15 out of 37 MPAL patients with available karyotypic data (40.5%) had complex karyotype. Complex karyotype that frequently appeared in MPAL was observed in lower incidence in Ly^+^AML (11/51, 21.6%) and Ly^−^AML (18/90, 20%). However, it seemed that MPAL patients had the lowest incidence of normal karyotype (27.0%) in the three groups (*P* = 0.003), and there were no statistical differences between the two groups of Ly^+^AML and Ly^−^AML. Philadelphia chromosome and fused BCR-ABL gene incidence were highest in MPAL group of patients (Table 1). Fluorescence in situ hybridization (FISH) examination was done in 34 MPAL patients, with details given in Table 1. Both MLL gene rearrangement and fused AML1/ETO gene were found to be positive in 1(2.9%) out of 34 cases analyzed, and BCR/ABL rearrangement was present in 7(20.6%) out of 34 cases.

### 3.4. Comparison of Various Immunophenotype on Survival Outcomes

**Figure 1:**
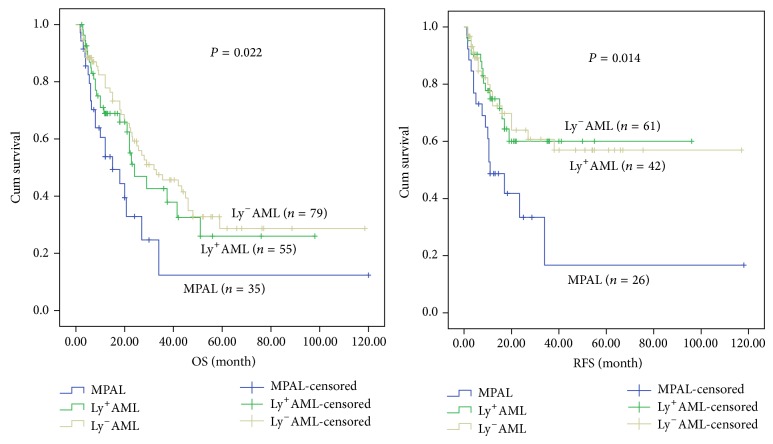
Kaplan-Meier plots for overall and relapse-free survival of the whole MPAL, Ly^+^AML, and Ly^−^AML patients.

Patients who received at least 2 circles of chemotherapy were involved in current study in order to analyze clinic outcome. The overall survival (OS) and relapse-free survival (RFS) rates for 35 MPAL, 55 Ly^+^AML, and 79 Ly^−^AML patients according to WHO 2008 criteria were shown in Table 2 and Figure 1. The OS and RFS were varied significantly among the three groups according to WHO 2008 classification (*P* = 0.022 and *P* = 0.014). By applying the results of paired comparison, we found that the OS in MPAL was significantly shorter than that in Ly^+^AML (*P* = 0.046) and Ly^−^AML patients (*P* = 0.006). Meanwhile, the RFS in MPAL was also significantly shorter than that in Ly^+^AML (*P* = 0.019) and Ly^−^AML group (*P* = 0.007). However, the OS and RFS were not significantly different between Ly^+^AML and Ly^−^AML groups (Table 2 and Figure 1).

### 3.5. Treatment Response and Outcome

In Ly^+^AML and Ly^−^AML groups with normal karyotype, complete remission (CR) rates were 88.5% (23/26) and 80.4% (37/46) after a second induction regimen, which was higher than the cases with complex karyotype (*P* = 0.013 and *P* = 0.029). However, in MPAL group, there was no difference in CR rate between normal karyotype and complex karyotype patients (77.8% versus 46.2%, *P* = 0.138). Meanwhile, the overall final complete remission rate after two cycles of chemotherapy of AML patients with different immunophenotype (MPAL, Ly^+^AML, and Ly^−^AML) had no statistical differences in the same genetic background. Details were shown in Table 3.

**Figure 2:**
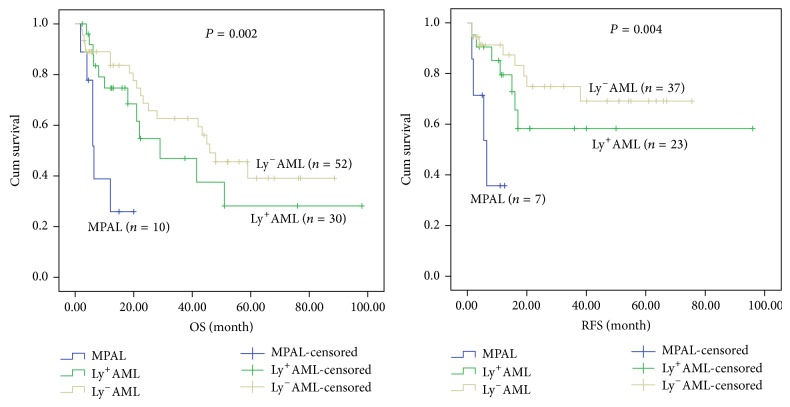
Kaplan-Meier plots for overall and relapse-free survival of AML patients with the normal karyotype established according to immunophenotype.

**Figure 3:**
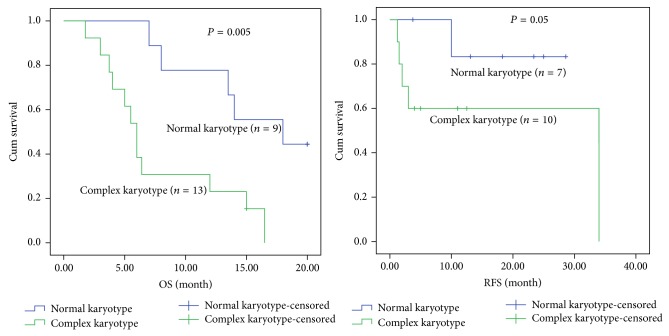
Kaplan-Meier plots for overall and relapse-free survival of MPAL patients established according to the normal and complex karyotype.

**Figure 4:**
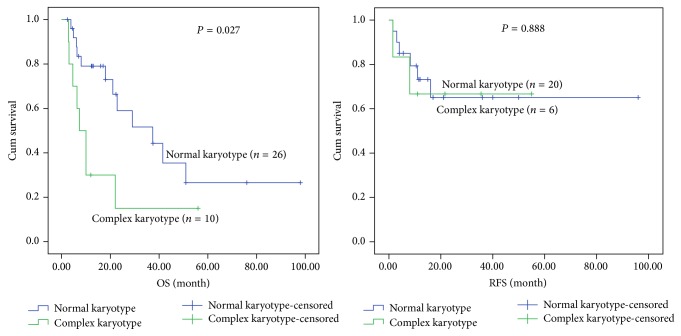
Kaplan-Meier plots for OS and RFS for Ly^+^AML patients with the normal and complex karyotype.

**Figure 5:**
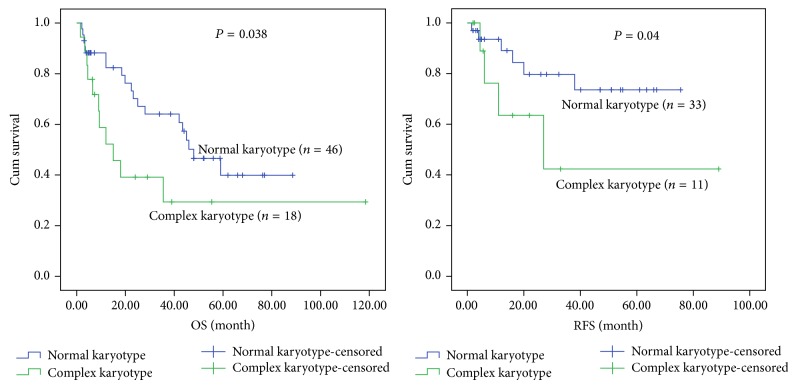
Kaplan-Meier plots for OS and RFS for Ly^−^AML patients with the normal and complex karyotype.

When only patients with normal karyotype were considered, MPAL patients appeared to have low median survival time (6.4 months) compared with Ly^+^AML (29 months) and Ly^−^AML (46 months) patients. There was significant difference in overall and relapse-free survival rates among MPAL, Ly^+^AML, and Ly^−^AML patients with normal karyotype (*P* = 0.002 and *P* = 0.004). By applying the results of paired comparison, the OS rate of MPAL patients was significantly lower than that of the Ly^+^AML (*P* = 0.008) and Ly^−^AML (*P* = 0.001), but no statistical differences were found between Ly^+^AML and Ly^−^AML patients. The RFS in MPAL with normal karyotype was significantly lower than that in the Ly^+^AML (*P* = 0.02) and Ly^−^AML (*P* = 0.002), but there was no statistical difference between the latter two groups, as shown in Figure 2. With regard to the patients with complex karyotype (except for the cases with Ph^+^ chromosome or BCR/ABL fusion gene), there were no statistical differences among the 3 groups in OS and RFS. The median survival time of patients with complex karyotype in three groups was 7.5 months in MPAL, 9.4 months in Ly^+^AML, and 15 months in Ly^−^AML, respectively (Figures 3, 4, and 5).

The OS and RFS rates of MPAL, Ly^+^AML, and Ly^−^AML patients with either normal or complex karyotype were shown, respectively, in Figures 3, 4, and 5. Except for the Ly^+^AML group, the RFS rates of MPAL and Ly^−^AML patients with complex karyotype were lower than those in patients with normal karyotype (*P* = 0.05 and *P* = 0.04). There were statistical differences of OS between MPAL patients with normal and complex karyotype (*P* = 0.005). Meanwhile, MPAL patients with normal karyotype had distinctly longer median survival time than those with complex karyotype (median 18 months versus 6 months). Furthermore, statistical differences were also found in the Ly^+^AML and Ly^−^AML patients between normal and complex karyotype (*P* = 0.027 and *P* = 0.038). In the two groups (Ly^+^AML and Ly^−^AML), median survival time was 37.4 and 46 months for patients with normal karyotype versus 7.3 and 15 months for patients with complex karyotype (Figures 3, 4, and 5).

In order to clarify whether the parameters with impact on survival added information to other known prognostic factor, analysis of the relative value of the prognostic factors for outcome were based on Cox's proportional hazards regression models for DFS and OS. In univariate analysis, 6 variables were included (age, gender, peripheral white blood cells, blasts in bone marrow, chromosome karyotype, and immunophenotype) to attempt identifying risk factors relating to OS. Age, peripheral white blood cells, MPAL, complex karyotype, and abnormal karyotype were shown to display adverse prognostic significance on OS (Table 4). However, in multivariate analysis for OS, only peripheral white blood cells (WBC) and complex karyotype maintained its deleterious independent factor (Table 4). Meanwhile, when analysis was performed in the overall patients, MPAL (*P* < 0.05) and complex karyotype (*P* < 0.01) in univariate analysis and complex karyotype (*P* < 0.01) in multivariate analysis were also poor prognostic factors on RFS (Tables 4 and 5). So it was showed that complex karyotype was an independent pejorative factor for both OS and RFS.

## 4. Discussion

AML is a genetically heterogeneous disease with widely different treatment outcomes and disparate survivals. Thus, it is important to propose prognostic model effectively for subtype of AML subtypes diagnosis and evaluations of outcome. Now, the most important prognostic factors for AML are age and chromosome karyotype [9, 10]. However, although patients have the same age and chromosome karyotype, the prognosis varies due to other factors, such as mixed phenotype acute leukemia (MPAL). For classification of biphenotypic acute leukemia, several classification systems were released including EGIL1998 criteria. Although in the last decade, EGIL1998 scoring system was used widely in clinical practice, limitation still existed because diagnosis of AL subtypes strictly according to immunophenotype did not include morphocytology, cytochemistry, and cytogenetics. Also, importantly, another limitation was when immunophenotyping was done only by flow cytometry; it was difficult to rule out the integral deviation caused by false negative and positive. In 2008, new WHO classification system for classification of MPAL was proposed. In the new scoring system, many parameters such as the flow cytometry, immunohistochemical staining, cytochemical staining, and immune-electron microscopy were used in order to diagnose AL subtypes [11, 12].

According to the WHO 2008 classification of leukemia [8], MPAL is an independent and uncommon entity. Hitherto, only two largest MPAL series which followed the WHO 2008 criteria have been published, which include the Chinese experience published by Yan et al. in 2012 with 117 patients [4] and the European series published by Matutes et al. in 2011 [12]. Here, we conducted a retrospective chart review of 47 MPAL patients according to these criteria. Similar to previous reports [7, 12], the proportion of blasts in bone marrow and the incidence of invasion in lymph node, liver, and spleen in MPAL group was significantly higher than that in Ly^+^AML and Ly^−^AML groups. And, in the current study, the median age of 34 years at diagnosis and a slight predominance of the male population (63.8%) is similar to that reported by Yan et al. [4]. Our results also showed a high incidence of Philadelphia chromosome positive (21.6%) or BCR-ABL translocation (20.6%) in MPAL, which was consistent with the report by Atfy et al. [13]. As reported by Matutes et al. [12] and Bachir et al. [14], we also observed a high incidence of cytogenetic abnormalities in MPAL with only a small number of the cases displaying a normal karyotype.

The previous studies showed that survival in biphenotypic acute leukemia was shorter than in other acute leukemia patients whatever adults or children [6, 7, 15–17]. According to our data, there were distinct OS and RFS differences among MPAL, Ly^−^AML, and Ly^+^AML. However, there were no significant differences in OS and RFS between Ly^−^AML and Ly^+^AML patients. These results demonstrated that MPAL may be a poor prognostic indicator in AML. However, it needed to be taken with caution because of the high incidence of complex karyotype in MPAL patients included. Therefore, all patients with normal karyotype were considered only, and it was found that the survival of MPAL patients was also worse than that in Ly^+^AML and Ly^−^AML. Meanwhile, factors relating to survival were evaluated with the Cox proportional-hazards regression model. As a result, in multivariate analysis, only patients with complex karyotype predicted a shorter OS and RFS.

Notably, within the MPAL group, patients with complex karyotype had shorter survival compared with normal karyotype patients. Similar phenomena were also found on OS in Ly^+^AML and Ly^−^AML groups. These findings demonstrated that complex karyotype was strongly associated with adverse outcome in AML, which was in harmony with several early studies [18, 19]. It also indicated that, when combining mixed immunophenotype and complex karyotype, patients had the shortest OS and RFS. Interestingly, with regard to patients with complex karyotype only, there were no statistical differences among the 3 groups in OS and RFS. Why did Ly^+^AML and Ly^−^AML patients with complex karyotype present similar survival time with MPAL? A possible explanation could be that complex karyotype has a more significant impact on clinical outcome in AML patients with no matter which subtype of immunophenotype. Several studies also demonstrated that complex karyotype is a poor prognosis factor [18, 19], and complex karyotype has a high frequency of P53 mutation, which may be associated with poor outcome in AML [19]. However, the information of complex karyotype in MPAL is limited because of the rarity of cases. Larger prospective studies are required to confirm the findings presented in this study. Though there were lots of studies about outcome when stratifying patients according to immunophenotype or cytogenetic abnormalities, none of these studies analyzed immunophenotyping on the basis of different genetic features in the prognostication.

In multivariate analysis for OS, in addition to complex karyotype, peripheral white blood cells (WBC) were also found as risk factor as it was also established in report by Mikulic et al. [20]. However, all other investigated clinical and biological parameters did not show any significant differences between Ly^+^AML and Ly^−^AML, which was consistent with those of Casasnovas et al. [21]. To our knowledge, though there were many studies comparing MPAL patients with AML patients, this is the first report about the analysis of clinical feature and prognosis outcome between MPAL and Ly^+^AML patients.

In conclusion, MPAL is confirmed to be a poor-risk acute leukemia subtype with normal chromosome karyotype, while Ly^+^AML does not impact prognosis. Complex karyotype is an unfavorable prognostic factor in AML patients with different immunophenotype. Moreover, mixed immunophenotype and complex karyotype increase the adverse risk when they coexist.

## Figures and Tables

**Table 1:** Clinical features and biological characteristics of patients with MPAL, Ly^+^AML, and Ly^−^AML [*n* (%)].

	MPAL	Ly^+^AML	Ly^−^AML	*P* value
Total number of cases	47	60	90	
Age (y)				
Median	34	34.5	39.5	NS
Range	3~72	2~83	3~84	
Sex				
Male	30 (63.8)	33 (55)	55 (61.1)	NS
Female	17 (36.2)	27 (45)	35 (38.9)	
WBC (×10^9^/L)				
Median	20.16	30.6	24.1	NS
Range	0.8~620	1.02~337.3	1~440.98	
Blasts in BM (%)				
Median	67.6	45.8	55.7	0.01
Range	28~90	20.8~88.5	20.42~95	
Hepatosplenomegaly and lymphadenectasis	30 (63.8)	26 (43.3)	36 (40.0)	0.024
Immunophenotyping				
CD34+	36 (76.6)	49 (81.7)	43 (47.8)	<0.001
CD117+	4 (8.5)	37 (61.7)	46 (51.1)	<0.001
HLA-DR+	36 (76.6)	51 (85.0)	67 (74.4)	NS
Karyotypic analysis (*n*)	37	51	90	
Normal	10 (27.0)	30 (58.8)	52 (57.8)	0.003
Complex	15 (40.5)	11 (21.6)	18 (20.0)	0.042
Abnormal	12 (32.4)	10 (19.6)	20 (22.2)	NS
Ph^+^	8 (21.6)	0 (0)	0 (0)	<0.001
FISH detection (*n*)	34	53	73	
BCR/ABL(+)	7 (20.6)	1 (1.9)	0 (0)	<0.001
AML1-ETO(+)	1 (2.9)	11 (20.8)	11 (15.1)	NS
MLL(+)	1 (2.9)	1 (1.9)	2 (2.7)	NS

*Note.* (1) Cases with chronic myeloid leukemia or myelodysplastic syndrome, acute promyelocytic leukemia, and secondary leukemia were excluded in this paper. (2) One-way ANOVA was applied for comparison measurement data like onset age and percentage of leukemia cells in BM and white blood cell count among the 3 groups. Chi-square test was used for comparison of other proportion. NS: no significance. WBC: white blood cell.

**Table 2:** Response to therapy in MPAL, Ly^+^AML, and Ly^−^AML patients [*n* (%)].

Group	*N*	Induction regimens/CR			CR after 2 courses	Postremission therapy			Relapse rate	Median time (Months)	
		①	②	③		④	⑤	⑥		RFS	OS
MPAL	35	15 (42.8)/12 (80.0)	14 (40.0)/11 (78.6)	6 (17.1)/3 (50.0)	26 (74.2)	6 (23.1)	7 (26.9)	13 (50.0)	11 (42.3)	11	15
Ly^+^AML	55	3 (5.5)/3 (100)	0/0	52 (94.5)/39 (75.0)	42 (76.4)	11 (26.2)	24 (57.1)	7 (16.7)	14 (33.3)	15	24
Ly^−^AML	79	0/0	0/0	79 (100)/60 (75.9)	60 (75.9)	26 (43.3)	18 (30.0)	16 (26.7)	17 (28.3)	20	32
*P* value	—	—	—	NS	NS	—	—	—	NS	<0.05^*^	<0.05^*^

*Note.* ① Induction treatment with combined AML/ALL drugs: DOALP (D: daunorubicin, O: vincristine, A: cytarabine, L: L-asparaginase, and P: prednisone) and DOAP. ② ALL-type induction therapy: VDLP (V: vincristine) and VDCP (c: cyclophosphamide). ③ AML-type induction therapy: DA/DAE/HA/TA (E: etoposide, H: homoharringtonine, and T: pirarubicin). ④ 3 courses of consolidation therapy and less than 3 courses of MD-Ara-C/HD-MTX. ⑤ Equal to or more than 3 courses of MD-Ara-C/HD-MTX or more than 3 courses of hyper-CVAD-A/B or autotransplantation after 5 median courses of standard-dose induction therapy, which ranges from 3 to 10 courses. ⑥ Allogeneic hematopoietic stem cell transplantation after 3–6 courses of consolidation or intensive therapy.

^*^Statistical significant difference among MPAL, Ly^+^AML, and Ly^−^AML groups.

NS: no significance among MPAL, Ly^+^AML, and Ly^−^AML groups.

**Table 3:** CR rate in MPAL, Ly^+^AML, and Ly^−^AML patients with different chromosome karyotype [*n* (%)].

	Normal karyotype		Complex karyotype		*P*2
	*N*	CR	*N*	CR	
MPAL	9	7 (77.8)	13^*^	6 (46.2)	NS
Ly^+^AML	26	23 (88.5)	10	5 (50.0)	0.013
Ly^−^AML	46	37 (80.4)	18	9 (50.0)	0.029
*P*1	—	NS	—	NS	—

*Note. N*: number of patients who received at least 2 circles of chemotherapy with normal or complex karyotype; ^*^except for one case with Ph^+^ chromosome; *P*1: the statistical comparison results of therapeutic response among MPAL, Ly^+^AML, and Ly^−^AML groups under the same genetic background; *P*2: the statistical comparison results of therapeutic response among MPAL, Ly^+^AML, and Ly^−^AML groups under different genetic background. NS: no significance.

**Table 4:** Univariate Cox model for OS and RFS risk factors of MPAL, Ly^+^AML, and Ly^−^AML patients.

Risk factors	OS			RFS		
	HR	95% CI	*P* value	HR	95% CI	*P* value
Age	1.014	1.001–1.027	0.031	1.013	1.000–1.026	0.055
Gender	1.023	0.68–1.540	0.912	1.150	0.761–1.737	0.507
WBC	1.003	1.001–1.006	0.012	1.002	1.000–1.004	0.102
BM blast	0.996	0.986–1.006	0.429	0.996	0.986–1.006	0.452
Chromosome karyotype						
Normal	1.000	—	—	1.000	—	—
Complex	2.409	1.429–4.060	0.001	2.691	1.591–4.553	0.000
Abnormal	1.808	1.018–3.211	0.043	1.718	0.973–3.031	0.062
Immunophenotype						
Ly^−^AML	1.000	—	—	1.000	—	—
Ly^+^AML	1.404	0.768–2.565	0.270	0.808	0.444–1.472	0.486
MPAL	1.446	0.905–2.312	0.023	1.063	0.600–1.545	0.046

HR: hazard ratio; CI: confidence interval; WBC: white blood cell; OS: overall survival; RFS: relapse-free survival.

**Table 5:** Multivariate Cox model for OS and RFS risk factors of MPAL, Ly^+^AML, and Ly^−^AML patients.

Risk factors	OS			RFS		
	HR	95% CI	*P* value	HR	95% CI	*P* value
WBC	1.003	1.001–1.006	0.009	—	—	—
Chromosome karyotype						
Normal	1.000	—	—	1.000	—	—
Complex	2.068	1.284–3.330	0.003	2.140	1.331–3.441	0.002
Abnormal	1.735	0.995–3.023	0.052	1.498	0.865–2.594	0.149

HR: hazard ratio; CI: confidence interval; WBC: white blood cell; OS: overall survival; RFS: relapse-free survival.
